# Short-term fertilizer application alters phenotypic traits of symbiotic nitrogen fixing bacteria

**DOI:** 10.7717/peerj.1291

**Published:** 2015-10-08

**Authors:** Anna K. Simonsen, Shery Han, Phil Rekret, Christine S. Rentschler, Katy D. Heath, John R. Stinchcombe

**Affiliations:** 1 Department of Ecology and Evolutionary Biology, University of Toronto, Toronto, Canada; 2 Department of Integrative Biology, University of Guelph, Guelph, Canada; 3 Department of Plant Biology, University of Illinois at Urbana-Champaign, Urbana, IL, United States of America; 4 Centre for the Analysis of Genome Evolution and Function, University of Toronto, Toronto, Canada

**Keywords:** Rhizobia, Mutualism, Fertilizer, Quantitative genetics, Partner quality, Nitrogen fixing bacteria, Plasticity, Nutrients, Legume, Host

## Abstract

Fertilizer application is a common anthropogenic alteration to terrestrial systems. Increased nutrient input can impact soil microbial diversity or function directly through altered soil environments, or indirectly through plant-microbe feedbacks, with potentially important effects on ecologically-important plant-associated mutualists. We investigated the impacts of plant fertilizer, containing all common macro and micronutrients on symbiotic nitrogen-fixing bacteria (rhizobia), a group of bacteria that are important for plant productivity and ecosystem function. We collected rhizobia nodule isolates from natural field soil that was treated with slow-release plant fertilizer over a single growing season and compared phenotypic traits related to free-living growth and host partner quality in these isolates to those of rhizobia from unfertilized soils. Through a series of single inoculation assays in controlled glasshouse conditions, we found that isolates from fertilized field soil provided legume hosts with higher mutualistic benefits. Through growth assays on media containing variable plant fertilizer concentrations, we found that plant fertilizer was generally beneficial for rhizobia growth. Rhizobia isolated from fertilized field soil had higher growth rates in the presence of plant fertilizer compared to isolates from unfertilized field soil, indicating that plant fertilizer application favoured rhizobia isolates with higher abilities to utilize fertilizer for free-living growth. We found a positive correlation between growth responses to fertilizer and mutualism benefits among isolates from fertilized field soil, demonstrating that variable plant fertilizer induces context-dependent genetic correlations, potentially changing the evolutionary trajectory of either trait through increased trait dependencies. Our study shows that short-term application is sufficient to alter the composition of rhizobia isolates in the population or community, either directly though changes in the soil chemistry or indirectly through altered host legume feedbacks, and is potentially a strong selective agent acting on natural rhizobia populations.

## Introduction

One of the largest human impacts on terrestrial ecosystems has been the widespread application of fertilizer for agricultural purposes. Ecosystem processes are well known to be altered by fertilizer input, including biogeochemical cycles (nitrogen and phosphorus), greenhouse gas emissions (Smith & Conen, 2004) and plant diversity and productivity. Soil bacteria and fungi have a major role in mediating terrestrial ecosystem processes (Van Der Heijden, Bardgett & Van Straalen, 2008; Wall & Moore, 1999) and increasing evidence has shown that soil fertilization affects microbial diversity, abundance and function (Marschner, Kandeler & Marschner, 2003; Sarathchandra et al., 2001; Sessitsch et al., 2001; Yu et al., 2015). Increasing evidence of correlated spatial and temporal shifts in community composition of soil microbes and plants due to complex direct and indirect feedbacks (Kardol et al., 2007; Bever, Platt & Morton, 2012) also suggest that fertilizer application can alter complex feedbacks between belowground and aboveground ecological processes. To date, the majority of studies measure microbial traits at an aggregated level—the entire population or community. Despite the potential for rapid evolutionary response to selective pressures from anthropogenic activity, few studies have measured individual-level phenotypes to investigate shifts in the mean trait value between populations experiencing different selective environments—an important step in documenting evolutionary responses (but see Weese et al., 2015). In this study, we measured various phenotypic traits at the individual/isolate level to examine whether fertilizer application causes shifts in traits of rhizobia, a functionally important bacterial group that play a major role in nitrogen cycling and plant growth through biological nitrogen-fixation.

Legumes, like most terrestrial plants, are nitrogen limited in most environments. Rhizobia fix atmospheric nitrogen and make it available to leguminous host plants through a mutualistic symbiotic association. While it is now common knowledge that legumes (and all other plants) respond to nutrient addition, the effects of fertilizer on rhizobia are not as well understood. Previous studies have firmly established that legumes suppress associations with rhizobia as plant-available nitrogen increases (Streeter & Wong, 1988; Imsande, 1986; Carroll & Gresshoff, 1983), and nitrogen addition has been shown to decrease rhizobia abundance (Coelho et al., 2009). However, fertilizer inputs typically contain combinations of macronutrients (i.e., N, P, K) and micronutrients (i.e., Mg, Fe) to enhance plant productivity, which may have different effects on legume-rhizobia interactions than variable nitrogen alone. An increase in rhizobia abundance from fertilizer application (Yan et al., 2014; Germida, 1988) suggests that combined nutrient addition could stimulate rhizobia growth due to the availability of elements that would ordinarily limit free-living cellular growth in the soil (O’hara, Boonkerd & Dilworth, 1988). Alternatively, rhizobia growth could be increased as a result of increased availability of other nutrients (i.e., phosphorus) that stimulate rhizobia root associations on legume roots (Gates & Wilson, 1974; Israel, 1987; Asimi, Gianinazzi-Pearson & Gianinazzi, 1980). Generally, these observations suggest that fertilizer addition could be an important selective force on natural rhizobia populations and may cause phenotypic changes in traits that are relevant to the free-living persistence and legume symbiosis of rhizobia.

Variation in traits is a ubiquitous property among rhizobia strains and has important consequences for understanding the impacts of ecological and evolutionary processes of altered soil environments on indigenous rhizobia populations. Nutrient addition could act directly on trait variation related to free-living vigour or growth by selecting traits that can tolerate higher ranges of nutrient input. Nutrient addition could also select traits related to mutualistic association as a result of altered host feedback responses. For example, long term nitrogen addition has been shown to reduce the mutualistic benefit of rhizobia isolates towards their legume hosts (Weese et al., 2015), an evolutionary response that could be caused by various mechanisms, including reduced selective pressure from legumes to maintain beneficial partners (Kiers, Hutton & Denison, 2007). Phenotypic changes in traits related to fertilizer tolerance or mutualism benefit could also occur as a result of selection on genetically correlated traits. For example, if traits related to fertilizer tolerance during free-living stages are genetically correlated with mutualism benefit traits through linkage- disequilibrium, changes in mutualism benefit could occur indirectly as a result of selection acting on free-living growth and persistence in the soil (Sachs, Russell & Hollowell, 2011). Conversely, if selection alters mutualism benefit traits, a genetic correlation would indirectly alter traits related to fertilizer tolerance during free-living growth. Therefore, genetic correlations between traits related to free-living growth and mutualism benefit are important in identifying additional evolutionary pathways that result in phenotypic changes in either trait.

In this study, we use a variety of approaches to investigate the effects of plant fertilizer (containing all macro and micro-nutrients) on phenotypic traits relevant for fitness of nitrogen-fixing rhizobia symbionts using a legume common to agriculturally disturbed systems, *Medicago lupulina*. We first applied fertilizer over a single growing season and then tested for community-level differences between fertilized and unfertilized soils on *M. lupulina* performance using whole-soil inoculations in the glasshouse. Next we disentangled the effects of rhizobia populations from those of the rest of the soil community by culturing rhizobia isolates and comparing and correlating *in vitro* free-living growth and mutualism benefit (i.e., host partner quality) of individual rhizobia isolates from either fertilized or unfertilized field soil. We further evaluated if isolates from fertilized or unfertilized field soil differed in their plastic responses to *in vitro* growth assays containing variable fertilizer concentrations.

## Methods

### Natural history of study system

The field experiment was conducted in a recently disturbed old field habitat with dense populations of the legume *Medicago lupulina* growing in the Koffler Scientific Reserve (www.ksr.utoronto.ca, 44.0300°N, 79.5275°W) in Southern Ontario, Canada. *M. lupulina* is an annual exotic that forms facultative mutualistic interactions with symbiotic nitrogen fixing bacteria, *Ensifer meliloti* and *Ensifer medicae* in loamy soils stereotypical of Southern Ontario soil profiles (Prévost & Bromfield, 2003; Bromfield et al., 2010). Interactions between *Medicago* and *Ensifer* occur in the early spring during plant germination when symbiotic bacteria infect plant roots and induce nodule formation. During nodule formation between *Medicago* and *Ensifer*, a portion of rhizobia cells differentiate into specialized cells that fix atmospheric nitrogen (Oke & Long, 1999). When plant seed set occurs in August and September, nodule plant tissue begins to senesce, releasing rhizobia cells into the soil—the undifferentiated fraction of the cells survive in a free-living state until the following growing season (Hirsch, 1996).

### Testing for field fertilizer treatment on host performance using whole-soil inoculations

We randomly positioned 16 plots (0.25 × 0.25 m) over two large *M. lupulina* population sites at the Koffler Scientific Reserve (44.0300°N, 79.5275°W). Within each population, half of the plots were randomly selected for fertilizer application. We applied 1 tbsp. (13 g) of Osmocote Miracle-Grow slow release fertilizer beads containing macro and micronutrients (in an N:P:K ratio of 19:6:12; Micromax^®^ by Scotts brand) over each plot in the early spring (May). When *Medicago lupulina* plants seeded and began to senesce by mid-August, soil was sampled at each plot and stored at 4 °C for further experimentation.

We initially tested for differential mutualistic effects of fertilized and unfertilized field soil on host plants by inoculating potted plants with whole soils in the glasshouse. We included three host genotypes (Cote-d’Or, France [FR]; Nebraska, USA [US] and Ontario, Canada [CA]; USDA germplasm repository: P1 234953-96i-SA19792, P1 215243-93i-53416, W6 4578-99i-2044) and a slow-release fertilizer application (same as field treatment) to determine whether the effects of field soil treatments were consistent across host genotype and fertilizer environment. In total, our design included field fertilizer treatment, glasshouse fertilizer treatment and host genotype in a full factorial design (i.e., 2 Field fertilizer treatments × 8 plots per field fertilizer treatment × 2 glasshouse fertilizer treatments per field plot × 3 plant genotypes per pot × 5 replicate pots = 480 total plants).

Each pot contained steam sanitized low nutrient soil (1:4; turface:sunshine mix #2) and a band of field soil applied (30 ml volume) at mid-depth and covered with a layer of autoclaved sand. To reduce effects on host performance as a result of chemical differences between fertilized and unfertilized field soil (as opposed to differences driven by microbial communities), we added autoclaved soil from the opposing field treatment to each pot in equal proportion. The opposing soil was prepared by autoclaving a soil mixture containing a subset of soil from all plots that received the same fertilizer treatment. For example, pots assigned with the unfertilized field soil treatment received 15 ml of unfertilized field soil from a given plot and 15 ml of autoclaved soil from all other fertilized field plots. We also added 10 control pots (which received 15 ml of autoclaved soil sample from each field treatment).

Prior to planting, seeds were scarified, sterilized in commercial bleach, stratified in the dark on 1.5% agar at 4 °C and pre-germinated at 22 °C for 12 h for radicle growth. Pre-germinated radicles from each host genotype were planted in each 6 inch pot (3 plant genotypes/pot). Plants were grown for 53 days and each pot was carefully top watered to minimize cross-contamination. At harvest, we recorded plant mortality, total dried plant biomass for each plant and haphazardly selected 15–20 nodules for dried preservation in tubes containing Drierite desiccant (Somasegaran & Hoben, 1994). Dried nodules were weighed to obtain a mean nodule mass measurement and stored at 4 °C in desiccant tubes for further experimentation. Plant performance was measured using plant biomass, which has been found to be strongly positively correlated with fruit and seed production in previous glasshouse experiments on *Medicago lupulina* in similar growing conditions (see Simonsen, Chow & Stinchcombe, 2014). Control plants showed significantly lower amounts of nodulation (11.2 nodules/plant) compared to experimental plants (127.2 nodules/plant; *t* = 8.6196, *p* < 0.0001), indicating that any low-level contamination that occurred is unlikely to explain experimental inoculation treatments.

We tested for field fertilizer treatments using a generalized linear mixed model on plant biomass, nodule size (log-transformed, PROC MIXED dist = Gaussian; SAS institute v9.3) and nodule number (PROC GLIMMIX, dist = Poisson). Our model included field fertilizer treatment, greenhouse fertilizer treatment, host genotype, block, final plant density in each pot (since some mortality occurred during the experiment), field site and harvest date as fixed effects, and pot and plot as random effects.

### Testing for field fertilizer treatment on host performance using single-isolate inoculations

We obtained individual rhizobia isolates using preserved nodules from the whole-soil inoculation and used them in a single-isolate inoculation experiment to provide a measure of each isolate’s mutualistic benefit towards its host and thus disentangle whole-soil effects from the rhizobia community on host traits. A preserved nodule was randomly selected from each unfertilized plant in the whole-soil inoculation experiment (described above), rehydrated in sterile water, and subcultured on Tryptone-Yeast (TY) agar media (Somasegaran & Hoben, 1994) until clean isolates were obtained. Field soil treatments that received the additional glasshouse fertilizer application were excluded from the rhizobia isolation procedure. A total of 191 isolates were successfully cultured (101 from unfertilized and 90 from fertilized field plots).

The US genotype was selected for use in this second experiment based on overall vigour, reduced mortality and since analysis showed no indication of host genotype*field fertilizer interaction in the initial experiment (see “Results”). Seeds were germinated as described above and planted in autoclaved turface:sunshine #2 mix (4:1) in 4 inch pots (1 plant/pot) in a randomized blocked glasshouse design (191 isolates × 5 replicate pots per isolate distributed over 5 blocks). Wild rhizobia isolates were grown in TY media for roughly 36–48 h and diluted to equalize cell inoculation densities (OD600 = 0.1; ∼10^6^ cells/ml); 5 ml of inoculant was applied to each pot. Preliminary culturing indicated that most isolates neared stationary phase of growth after 36 h. Plants were given nitrogen-free Fahraeus nutrient solution (Somasegaran & Hoben, 1994) once weekly until harvest at 80 days. We measured host performance as the sum of total fruit and flower production per individual plant at harvest. None of the un-inoculated control plants (*n* = 36) flowered at harvest and were also significantly smaller prior to harvest (5.7 leaves/plant compared to 26.8 leaves/plant on inoculated plants; *t* = 20.39, *p* < 0.0001).

We determined the field fertilizer effect on all measures of host performance using a generalized linear mixed model (PROC GLIMMIX, dist = over-dispersed Poisson for fruit and flower production; SAS institute v9.3). Field fertilizer treatment, genotype of origin host (where preserved nodule was obtained), origin field site, greenhouse block and harvest date were included as fixed effects, while field sample plot (of original field soil sample) was included as a random factor. We tested for isolate effects using a log-likelihood ratio test between the full mixed model containing isolate and reduced model excluding the isolate term.

### Testing for field fertilizer treatment on rhizobia isolate growth

We selected a random sub-set of isolates from each field fertilizer treatment for *in vitro* growth assays—31 from fertilized field soil (*n*_[CA]_ = 8, *n*_[FR]_ = 7, *n*_[US]_ = 16 from each host genotype) and 27 isolates from unfertilized field soil (*n*_[CA]_ = 8, *n*_[FR]_ = 11, *n*_[US]_ = 8. Isolates were grown in TY media containing three different plant fertilizer concentrations: no fertilizer (control), 0.25 tbsp. of fertilizer/500 ml media (low) and 0.5 tbsp. of fertilizer/500 ml media (high). All media was prepared using sterile filtered stock fertilizer solutions containing the same slow release fertilizer brand that was applied in the field plots (5 tbsp. of dissolved fertilizer per liter of distilled water). In total, our *in vitro* assay evaluated 58 isolates over 3 nutrient conditions with 8 replicates for each [isolate]*[fertilizer media] treatment combination.

We conducted the growth assays in 96 well plates, each containing 150 uL of liquid media and initially inoculated with 10 uL of diluted cell culture, grown initially in standard TY media for 36–48 h (OD_600_ = 0.1, roughly 10^6^ cells/ml). Initial cell density measurements were taken immediately following inoculation and at 36 h. Each plate assayed 4 isolates in all 3 media treatments (*n* = 8 wells/isolate in each media treatment), and 8 additional un-inoculated wells containing blank TY media. Isolates were randomly assigned over 6 trials and cell density was estimated by measuring optical density (OD_600_). We found no indication of contamination in un-inoculated controls (as indicated by unchanging optical density measurements during growth assays and a lack of cell growth when subsequently cultured on TY agar plates).

We evaluated the effect of field fertilization on optical density after 36 h using a generalized linear model (PROC MIXED, dist = Gaussian) containing field fertilizer, media fertilizer, origin host genotype and origin site as fixed effects. Optical density at initial inoculation was included as a covariate to account for any absorbance differences caused by fertilizer media treatment and initial inoculation. Isolate, trial, plate and field sample plot were included as random effects. We tested for isolate effects as above.

### Testing for associations between rhizobia growth and mutualism benefit traits

We calculated mean growth and host performance traits for each isolate using fixed- effect lsmeans from a mixed model output. We obtained means for cell density counts (at 36 h) across each fertilizer media treatment from a mixed model that included fertilizer media treatment and isolate as fixed effects, and trial and plate as random effects; lsmeans were obtained from [fertilizer media]*[isolate] term (PROC MIXED, dist = Gaussian). Additionally, we calculated a growth plasticity index as the ratio of growth response in fertilizer and no fertilizer after 36 h for each isolate (see ‘tolerance index’ in Thrall et al., 2009): [OD_high,36 h_ − OD_high,initial_]/(OD_control,36 h_ − OD_control,initial_] and [OD_low,36 h_ − OD_intermedite,initial_]/(OD_control,36 h_ − OD_control,initial_], where ‘control’, ‘low’ and ‘high’ refer to fertilizer concentration. For isolate means in host performance, total fruit and flower production was modelled by block, harvest date and isolate as fixed effects and lsmeans were obtained from the isolate term (PROC GLIMMIX, dist = over-dispersed Poisson). We tested for associations between *in vitro* growth assays and mutualistic benefit using a general linear model, with host performance as the response and cell density, field fertilizer treatment and host genotype as predictors. We repeated the model for each type of growth assay (cell density count in control, low and high nutrient and the growth plasticity index).

## Results

### Hosts performance in field soil inoculations

**Figure 1:**
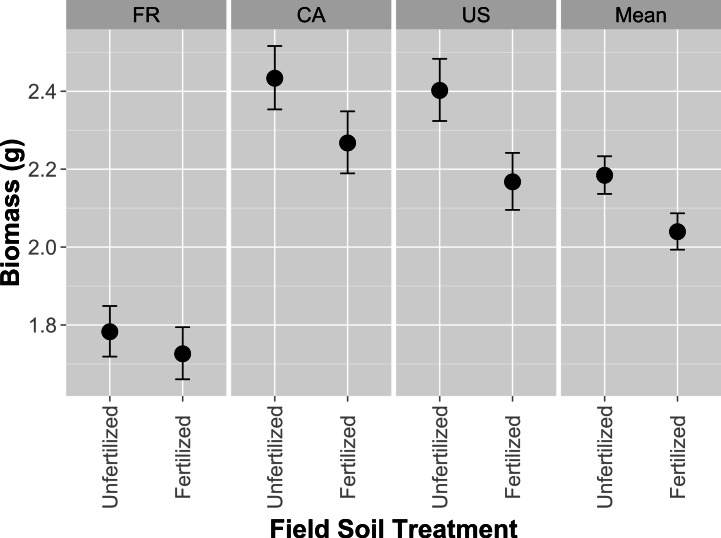
Host performance in whole-soil inoculations that have been fertilized or unfertilized in the field. Aboveground biomass (g) in *Medicago lupulina* when three plant genotypes (FR, CA and US) were inoculated with whole soil from field plots that were either fertilized with nutrients containing all conventional macro and micronutrients or remained unfertilized. Mean values for each field soil treatment combine glasshouse fertilized and glasshouse unfertilized treatments. Error bars represent standard errors.

Plant biomass was larger when hosts were grown in unfertilized field soil compared to fertilized field soil (Fig. 1; *F*_1,418_ = 471, *p* = 0.0306; Table S1). Expectedly plants were larger when fertilizer was applied to pots in the greenhouse (*F*_1,418_ = 121.36, *p* < 0.0001; Table S1). Host genotype also explained variation in plant size (Fig. 1; *F*_2,418_ = 42.47, *p* < 0.0001). However, host genotype and greenhouse fertilizer application did not alter rank effects of field fertilizer treatment, as indicated by a lack of interactive effects between the field and greenhouse fertilizer treatment (*F*_1,418_ = 0.7595, *p* = 0.7595; Table S1) and between field fertilizer and host genotype (*F*_2,418_ = 0.56, *p* = 0.5714). Nodule number and mean nodule size was 3.49% and 15.28% larger in fertilized field soil, but not significantly so (*F*_1,272_ = 2.73, *p* = 0.0997; *F*_1,13.11_ = 2.71, *p* = 0.1232 resp.). Generally, these results indicate that the higher host performance in unfertilized field soil was consistent across host genotypes and greenhouse fertilizer treatments.

### Hosts performance in single isolate inoculations

**Figure 2:**
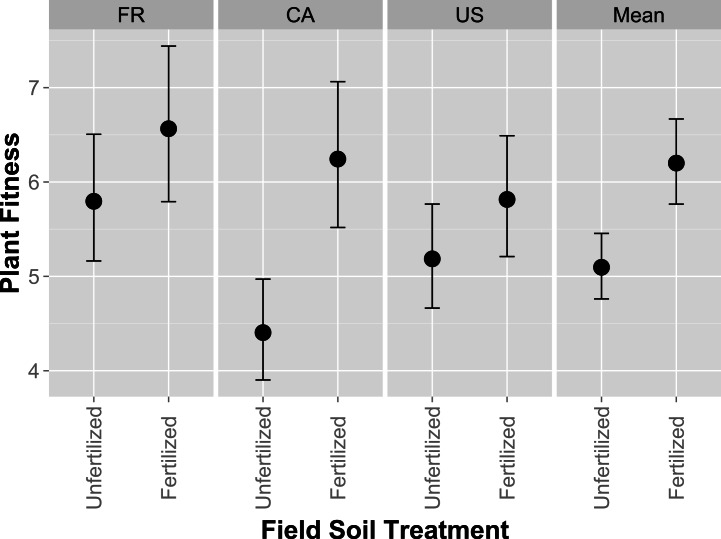
Host partner quality of rhizobia isolates from fertilized or unfertilized field soil using single-strain inoculations. Host partner quality, measured by plant fitness (total fruit and flower production), on the US plant genotype exposed to single-isolate inoculations of rhizobia isolated from fertilized or unfertilized treatments. FR, CA and US are host genotypes the isolates were originally cultured from. Mean values for each field soil treatment combine all isolates from every host genotype. Error bars represent standard errors.

Full model results are presented in Table S2. In contrast to whole-soil inoculation effects, plants had higher performance (measured by fruit and flower production) when inoculated with isolates originating from fertilized field soil (Fig. 2; *F*_1,152_ = 4.35, *p* = 0.0386). Biomass was non-significant, but trended in the same direction as fruit and flower production, being higher when inoculated with isolates from fertilized field soil (not shown). Host performance differed significantly among isolates (*χ*^2^ = 20.9, *p* < 0.0001), indicating genetic variation in rhizobia partner quality among our isolates. Rhizobia partner quality, or partner quality response to field fertilization, were not affected by the host genotype from which the isolate was originally collected (in the whole soil inoculum experiment), as indicated by non-significant Host genotype and Field Fertilizer*Host genotype effects (*F*_1,148.7_ = 1.01, *p* = 0.37 and *F*_1,148.7_ = 0.64, *p* = 0.5266; Table S2). These results indicate that isolates isolated from unfertilized field soil provided lower mutualism benefits to their host compared to isolates from fertilized field soil.

### Strain growth assays on differing media fertilizer concentrations

**Figure 3:**
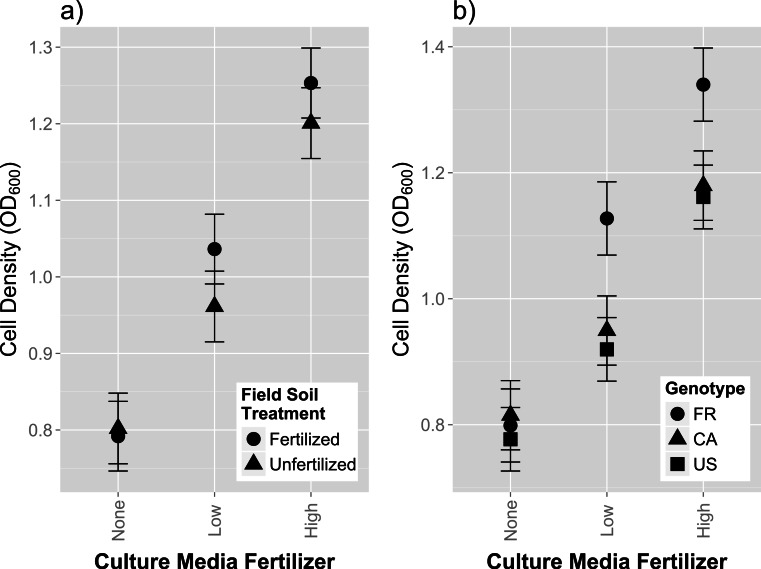
*In vitro* growth assays of rhizobia isolates in variable concentrations of fertilizer. Cell density, (measured by optical density at 600 nm), of single isolate rhizobia cultures grown in liquid media (after 36 h of growth) containing increasing concentrations of plant fertilizer. (A) Growth responses between isolates originally from fertilized and unfertilized field soil (B) Growth responses between isolates originally cultured from different host genotypes (FR, CA and US). Error bars represent standard errors.

We found no main effect of field fertilization on strain growth in culture (*F*_1,1118_ = 1.14, *p* = 0.2862; Table S3). Media fertilizer had a consistent and positive effect on rhizobia growth, causing intermediate cell density at low fertilizer concentrations, and high cell density counts at high media fertilizer concentrations (Fig. 3A, 3B and Table S3). We also found significant differences in growth among isolates (*χ*^2^ = 408.2, *p* < 0.0001; Table S3). Isolates originating from fertilized field soil exhibited the highest increase in growth as fertilizer media content increased, as indicated by a significant overall effect of [field fertilizer]^*^[media fertilizer] (Fig. 3A; *F*_2,1114_ = 4.08; *p* = 0.0172; Table S3). Host genotype origin also affected growth reaction norms, with isolates from FR hosts exhibiting the highest increase in growth across media fertilizer concentrations (Fig. 3B; *F*_4,1114_ = 9.40; *p* < 0.0001). These results indicate that field fertilizer treatment, rhizobia nodule isolates, and the genotype of the origin host all affect the degree of rhizobia plasticity in response to plant fertilizer in the liquid growth media.

### Correlations between free-living growth and mutualistic benefit

**Figure 4:**
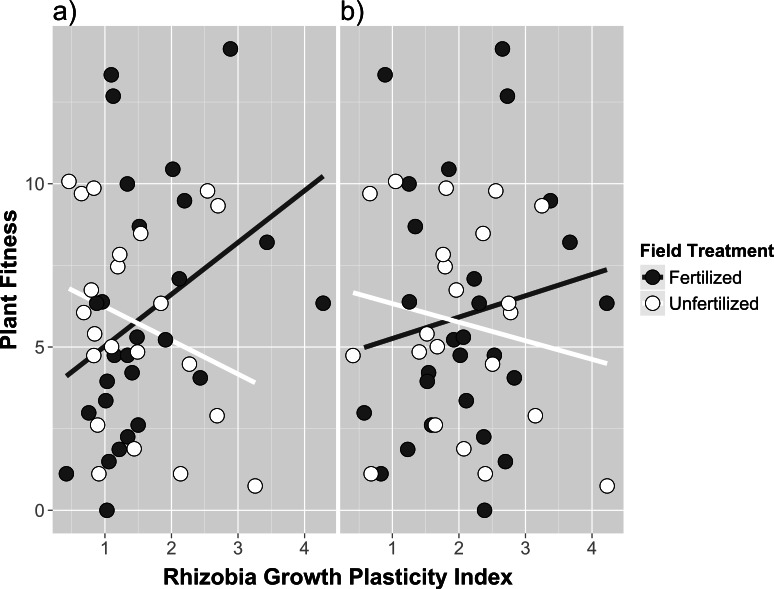
Genetic correlations between host partner quality and free-living growth of rhizobia isolates from fertilized and unfertilized field soil. Genetic correlation between host partner quality (measured as plant fitness using total fruit and flower production) and growth responses in rhizobia using growth plasticity indices calculated from (A) low media fertilizer concentrations and (B) high media fertilizer concentrations. Plasticity indices are the ratio of growth responses in fertilizer vs. no fertilizer after 36 h. Individual points are trait means calculated within each field and media fertilizer treatment for each rhizobia isolate.

We did not detect any broad association patterns between mutualistic benefit (measured by host performance) and cell growth measures using cell density estimates in any media fertilizer treatment (indicated by no significant main effect of cell density; see Table S4), nor did we detect any field fertilizer treatment specific associations between host performance and cell density estimated in any fertilizer culture media (indicated by no significant cell density*field fertilizer interaction; see Table S4). However, the low fertilizer growth plasticity index (the ratio of growth responses in low fertilizer vs. no fertilizer after 36 h) did show field treatment specific reaction norms, exhibiting a positive correlation between traits in the presence of field fertilizer and a negative correlation in unfertilized treatments (Fig. 4A; field fertilizer*growth plasticity index; *F*_1,43_ = 6.25, *p* = 0.0163 in Table S4). The high fertilizer growth plasticity index (the ratio of growth responses in high fertilizer vs. no fertilizer after 36 h) trended in similar but non-significant reaction norm patterns (Fig. 4B and Table S4).

## Discussion

We investigated how a single season of field fertilizer application in natural field soil altered phenotypic properties of symbiotic rhizobia that associate with *M. lupulina*. Our study shows that plant fertilizer changes host partner quality (as defined by host biomass, flower or fruit production) and free-living growth responses as well as environmentally dependent associations between these traits, demonstrating that long-term nutrient application across multiple years is not required to observe shifts in ecologically relevant phenotypic traits of symbiotic rhizobia populations.

### Growth responses of rhizobia to plant fertilizer

Our data show that, even after a single season of fertilizer application, isolates from fertilized field soil grew faster than isolates from unfertilized field soil when the growing media contained the original plant fertilizer used on field soil. These results suggests that field fertilizer application caused a community shift or within-species evolutionary change, favouring lineages or genotypes (or even alleles at specific genes) that are more capable of utilizing higher dosages of plant fertilizer for growth in the free-living state (in the soil or in culture) in more nutrient-rich conditions. Given that rhizobia isolates from both field soil treatments responded positively to fertilizer in agar media, indicating that plant fertilizer provide nutrients that ordinarily limit free-living growth, our data support the hypothesis that fertilizer affected rhizobia fitness components related to free-living persistence in the soil. However, we cannot exclude the possibility that fertilizer application affected symbiosis fitness components, favouring rhizobia isolates that are competitive for *Medicago* nodulation, thus gaining higher fitness through host feedbacks.

We found no preliminary evidence of a fitness trade-off for growth in higher fertilizer—rhizobia from fertilized field soil did not have lower growth rates than rhizobia from unfertilized field on control media containing no plant nutrients supplement (Fig. 3A). However, previous empirical studies have found fitness costs (affecting free-living persistence) to adaptation to salt (Thrall et al., 2009) and metal (Porter & Rice, 2012) in soil. It is possible that the fitness costs observed for higher metal and salt concentrations occurs because these factors are generally detrimental to rhizobia growth and may require physiological trade-offs for survival (i.e., minimizing uptake of harmful elements while maintaining acquisition of beneficial elements). A lack of a fitness cost observed in our experiment may be because plant fertilizer stimulates growth and thus does not require physiological trade-offs to survive in low or high nutrient conditions.

To our knowledge, this is the first study to provide evidence that plant fertilizer (containing all macro and micro nutrients) is directly beneficial for rhizobia growth and favours isolates that have higher growth response to fertilizer.

### Mutualism benefit responses of rhizobia to plant fertilizer

Initial whole field soil inoculations showed that *Medicago* had lower biomass in fertilized field soil. Since we expected higher biomass in fertilized field soil due simply to the presence of additional nutrients, these results suggest that plant fertilizer addition altered the microbial community composition in ways that are relevant to the aboveground productivity of *Medicago*. However, legume hosts were larger when inoculated directly with the rhizobia isolates cultured from fertilized soil, which suggests that the rhizobia community is unlikely to be responsible for the differences in plant performance observed in the initial whole-soil inoculations. In contrast, Weese et al. (2015) found the host partner quality decreased as a result of long term addition of nitrogen. We explored the possibility that the increase in host partner quality observed in our study may be due to a positive genetic correlation with free-living tolerance to fertilizer (i.e., isolates with higher mutualism benefit were observed due to a positive trait correlation with fertilizer tolerance). While we did detect a positive genetic correlation between host partner quality and plasticity in growth response to fertilizer, the direction of the correlation only occurred in fertilized field soil, being more strongly positive in the presence of fertilizer. The application of field fertilizer may have induced a positive correlation due to environment specific expression of genes that had pleiotropic effects on free-living growth and mutualism benefit traits, and may explain why both traits changed in our study. Generally, our data show that growth responses to fertilizer and mutualism benefit are not independent, and that the presence of fertilizer alters the degree of trait dependency for traits relevant to rhizobia fitness.

Another possible mechanism for increased host partner quality is that different host feedback dynamics occurred in our experiment compared to Weese et al. (2015). The addition of fertilizer containing excessive nitrogen (as the case with Weese et al., 2015) typically supresses associations with rhizobia, which is expected to alter host feedback dynamics by reducing fitness benefits towards beneficial symbiotic rhizobia. However, different host feedback dynamics may be induced if fertilizer contains increasing concentrations of all other macro macronutrients (i.e., N, P and K). For example, an increased nitrogen and phosphorus supply can increase symbiotic associations with beneficial rhizobia partners, which would then actually increase fitness benefits towards beneficial rhizobia partners.

Alternatively, fertilizer input in our experiment could have reduced selective pressure from plant feedbacks, which would cause host partner quality traits to increase by drift. However, replicated randomized plots allowed us to ascribe any differences among fertilizer types to the treatments we applied, making drift a less likely explanation. Furthermore, drift is expected to randomly exacerbate plot level differences between rhizobia populations, but plot had no explanatory effect in either host partner quality or *in vitro* growth traits in our study.

Further experimentation will be required to delineate the suite of causes that produced lower *Medicago* performance on the whole-soil inoculations and identify other microbial taxa relevant for plant productivity. It is possible that our experiment did not adequately capture the diversity of rhizobia present in the field soil. It is also possible that that strains occurring in mixtures have different effects on host fitness then single strain inoculations alone, which has been found in *Medicago* (Simonsen, Chow & Stinchcombe, 2014) and *Acacia* (Barrett et al., 2015). Decreased host productivity in fertilized whole soil inoculations could also result from changes in the abundance or diversity of beneficial symbiotic mycorrhizal fungi as a result of altered host feedbacks from excessive soil phosphorus addition (Broghammer et al., 2012). Previous experiments by Weese et al. (2015) and Thrall et al. (2007) also observed differences in whole-soil versus individual rhizobia isolate results on host condition and fitness. These studies, along with ours, highlight the challenge of focusing on individual taxa for inferring processes on complex non-additive effects of microbial communities on plant-microbe interactions, plant productivity and fitness.

### Host origin affects rhizobia traits

Legume hosts have an important influence on rhizobia fitness in soils. The particular host genotype and species also has an influence on rhizobia abundance and composition (Coelho et al., 2009). Our experiment further shows that the origin of the host genotype is also associated with colony growth phenotypes of individual rhizobia, regardless of the fertilizer treatment applied on the field soil (Fig. 3B). Specifically, we found that isolates from hosts originating from France (i.e., FR) had a larger response to growth on fertilized agar media compared to isolates obtained from hosts from Canada and United States. Interestingly, the French host performed the poorest in initial whole soil inoculation (Fig. 1). Isolates from the French host genotype also trended towards providing the highest mean mutualism benefit (Fig. 2). Our results suggest that the host genotype or the host genotype reaction to the soil affected the assemblage of symbiotic interacting rhizobia during the whole soil inoculation. Generally, these results are consistent with previous studies (Coelho et al., 2009; Heath & Tiffin, 2009; Lafay & Burdon, 2001; Hoque, Broadhurst & Thrall, 2011), which have found that host genotypes have strong effects on the assemblage of rhizobia isolates inhabiting the nodules and, therefore, subsequent effects on the genetic composition of the soil communities after plant senescence.

## Conclusion

Our experiments have shown that short-term application of plant fertilizer can select isolates that differ in phenotypic traits related to *in vitro* vigour and legume symbiosis, and together with fertilization responses in culture, suggest that the shift in growth responses was a result of a direct response to fertilizer application. However, the underlying causes of the observed increase in mutualism benefit as a result of fertilizer application will require further investigation with larger sub-samples of isolates, as the observed changes in trait values can still potentially be explained by altered host feedback responses. Our study importantly shows that fertilizer causes environment specific dependency between phenotypic traits, indicating that changes in growth and mutualism benefit traits will not act independently in response to fertilizer. Our findings support the emerging literature demonstrating that host genotypes, nutrient environments, and their interaction alter the phenotypic composition of natural rhizobia populations and, more generally, contributes to the nascent synthesis demonstrating important implications of anthropogenic disturbances on important mutualistic species interactions (Ratcliff, Kadam & Denison, 2008; Porter & Simms, 2014).

## Supplemental Information

10.7717/peerj.1291/supp-1 Table S1 Analysis on host partner quality (estimated as total dried plant biomass) when inoculated with fertilized or unfertilized field soil The effect of field fertilizer treatment on biomass was evaluated in a mixed model over a Gaussian distribution. Significance of random terms were evaluated using a log likelihood ratio test. Pot (random effect) and site (fixed effect) were excluded as neither explained any significant variation. Plant density within each pot was included to account for some plant mortality that occurred during the course of the experiment.

10.7717/peerj.1291/supp-2 Table S2 Analysis on host partner quality (estimated as total fruit and flower production) when inoculated with cultured isolates originating from fertilized and unfertilized field soil The effect of field fertilizer treatment on host performance was evaluated in a generalized linear mixed model over an over-dispersed Poisson distribution. Significance of random terms were evaluated using a log likelihood ratio test. Plot (random effect) and site (fixed effect) were excluded as neither explained any significant variation.

10.7717/peerj.1291/supp-3 Table S3 Analysis of isolate vigour (as measured by optical density OD_600_, an estimate of cell density) after 36 h of growth Nodule isolates originating from fertilized and unfertilized field soil (“Field Fertilizer” effect) were evaluated across three different liquid media (“Fertilizer in media” effect) containing either zero, low, or high levels of dissolved plant fertilizer. Host genotype refers to the genotype of the original host the isolate was isolated from after whole-soil inoculations. Initial cell density was included as a covariate to account for random differences in initial inoculation density at 0 h. Plot was excluded as a random effect, as it did not explain any variation.

10.7717/peerj.1291/supp-4 Table S4 Analysis of genetic correlations between host partner quality and *in vitro* cell growth responses Analysis of genetic correlations between host partner quality (estimated from total fruit and flower production) and *in vitro* cell growth responses using mean rhizobia isolate values. Rhizobia growth responses were measured using optical density readings (OD_600_) within each fertilizer concentration or a growth plasticity index (PI) across differing fertilizer concentrations (see methods for index calculation). Analysis was performed on trait means obtained from fixed-effect lsmean estimates from mixed models accounting for factors of non-interest (i.e., greenhouse block, cell culture plate). Mutualistic benefit was regressed against cell growth and the interaction between cell growth and fertilizer media treatments. Host genotype origin refers to the host genotype the isolate was isolated from during whole soil inoculations. Host genotype was included as genotype was identified as potentially important factor based on results of other components of this study. Non-significant interactive effects with Host genotype were excluded from the final model.

## Additional Information and Declarations

### Data Availability

The following information was supplied regarding data availability: Figshare: http://figshare.com/s/8e48d1ea544511e5a31306ec4bbcf141.
