# Supplementary material for: Short-term fertilizer application alters phenotypic traits of symbiotic nitrogen fixing bacteria
Source: PeerJ. 2015 Oct 8;3:e1291. doi: 10.7717/peerj.1291 (PMC4614912; doi:10.7717/peerj.1291)
Supplement: Table S1 — The effect of field fertilizer treatment on biomass was evaluated in a mixed model over a Gaussian distribution. Significance of random terms were evaluated using a log likelihood ratio test. Pot (random effect) and site (fixed effect) were excluded as neither explained any significant variation. Plant density within each pot was included to account for some plant mortality that occurred during the course of the experiment. [file peerj-03-1291-s001.docx]

Table S1: Analysis on host partner quality (estimated as total dried plant biomass) when inoculated with fertilized or unfertilized field soil. The effect of field fertilizer treatment on biomass was evaluated in a mixed model over a Gaussian distribution. Significance of random terms were evaluated using a log likelihood ratio test. Pot (random effect) and site (fixed effect) were excluded as neither explained any significant variation. Plant density within each pot was included to account for some plant mortality that occurred during the course of the experiment.

| Fixed Effects | F (NumDF, DenDF) | P | | |  |
| --- | --- | --- | --- | --- | --- |
| Field Fertilization (FF) | 4.71 (1,418) | 0.0306 | | |  |
| Greenhouse Fertilization (GF) | 121.36 (1,418) | <0.0001 | | |  |
| Host Genotype | 42.47(2, 418) | <0.0001 | | |  |
| FF*Host Genotype | 0.56 (2, 418) | 0.5714 | | |  |
| FF*GF | 0.09 (1,418) | 0.7595 | | |  |
| GF*Host Genotype | 4.88 (2, 418) | 0.0081 | | |  |
| FF*GF*Host Genotype | 1.01 (2, 418) | 0.3650 | | |  |
| Block | 14.64 (1,418) | 0.0001 | | |  |
| Plant density | 0.56 (1,418) | 0.4548 | | |  |
| Date of Harvest | 31.07 (1,418) | <0.0001 | | |  |
|  |  |  | | |  |
| Random Effects | Estimate | | | χ^2^ | P |
| Plot | 0.0010 ± 0.0015 | | 0.6 | | 0.2193 |
| Residual | 0.0794 ±0.0055 |  | | |  |
