# Supplementary material for: Short-term fertilizer application alters phenotypic traits of symbiotic nitrogen fixing bacteria
Source: PeerJ. 2015 Oct 8;3:e1291. doi: 10.7717/peerj.1291 (PMC4614912; doi:10.7717/peerj.1291)
Supplement: Table S2 — The effect of field fertilizer treatment on host performance was evaluated in a generalized linear mixed model over an over-dispersed Poisson distribution. Significance of random terms were evaluated using a log likelihood ratio test. Plot (random effect) and site (fixed effect) were excluded as neither explained any significant variation. [file peerj-03-1291-s002.docx]

Table S2: Analysis on host partner quality (estimated as total fruit and flower production) when inoculated with cultured isolates originating from fertilized and unfertilized field soil. The effect of field fertilizer treatment on host performance was evaluated in a generalized linear mixed model over an over-dispersed Poisson distribution. Significance of random terms were evaluated using a log likelihood ratio test. Plot (random effect) and site (fixed effect) were excluded as neither explained any significant variation.

| Fixed Effects | F (NumDF, DenDF) | P |  |
| --- | --- | --- | --- |
| Field Fertilization (FF) | 4.35 (1, 152) | 0.0386 |  |
| Host Genotype | 1.01 (2, 147.8) | 0.3668 |  |
| FF*Genotype | 0.64 (1, 148.7) | 0.5266 |  |
| Site | 0.69 (1, 145.9) | 0.4070 |  |
| Block | 12.18 (4, 767.7) | <0.0001 |  |
| harvest date | 253.36 (1, 888.8) | <0.0001 |  |
|  |  |  |  |
| Random Effects | Estimate | χ^2^ | P |
| Isolate | 0.1772 ± 0.0468 | 20.9 | <0.00001 |
| Residual | 3.8761 ± 0.2059 |  |  |
