# Supplementary material for: Short-term fertilizer application alters phenotypic traits of symbiotic nitrogen fixing bacteria
Source: PeerJ. 2015 Oct 8;3:e1291. doi: 10.7717/peerj.1291 (PMC4614912; doi:10.7717/peerj.1291)
Supplement: Table S3 — Nodule isolates originating from fertilized and unfertilized field soil (“Field Fertilizer” effect) were evaluated across three different liquid media (“Fertilizer in media” effect) containing either zero, low, or high levels of dissolved plant fertilizer. Host genotype refers to the genotype of the original host the isolate was isolated from after whole-soil inoculations. Initial cell density was included as a covariate to account for random differences in initial inoculation density at 0 h. Plot was excluded as a random effect, as it did not explain any variation. [file peerj-03-1291-s003.docx]

Table S3: Analysis of isolate vigour (as measured by optical density OD_600_, an estimate of cell density) after 36 hours of growth. Nodule isolates originating from fertilized and unfertilized field soil (“Field Fertilizer” effect) were evaluated across three different liquid media (“Fertilizer in media” effect) containing either zero, low, or high levels of dissolved plant fertilizer. Host genotype refers to the genotype of the original host the isolate was isolated from after whole-soil inoculations. Initial cell density was included as a covariate to account for random differences in initial inoculation density at 0 hours. Plot was excluded as a random effect, as it did not explain any variation.

| Fixed Effects | F (NumDF, DenDF) | P |  |
| --- | --- | --- | --- |
| Field Fertilization (FF) | 1.14 (1, 1118) | 0.2862 |  |
| Fertilizer in media (MF) | 377.27 (2, 1118) | <0.0001 |  |
| Host Genotype | 1.20 (2, 1118) | 0.3013 |  |
| FF*MF | 4.08 (2, 1118) | 0.0172 |  |
| MF*Host Genotype | 9.40 (4, 1118) | <0.0001 |  |
| FF*Genotype | <0.00 (2, 1114) | 0.9979 |  |
| Site | 2.50 (1, 1114) | 0.1143 |  |
| Initial cell density | 0.40 (1, 1114) | 0.5279 |  |
|  |  |  |  |
| Random Effects | Estimate | χ^2^ | p |
| Trial | 0.0022 ± 0.0038 | 0.6 | 0.2193 |
| Isolate | 0.0298 ± 0.00717 | 408.6 | <0.00001 |
| Residual | 0.0418 ± 0.00177 |  |  |
