# Supplementary material for: Short-term fertilizer application alters phenotypic traits of symbiotic nitrogen fixing bacteria
Source: PeerJ. 2015 Oct 8;3:e1291. doi: 10.7717/peerj.1291 (PMC4614912; doi:10.7717/peerj.1291)
Supplement: Table S4 — Analysis of genetic correlations between host partner quality (estimated from total fruit and flower production) and in vitro cell growth responses using mean rhizobia isolate values. Rhizobia growth responses were measured using optical density readings (OD600) within each fertilizer concentration or a growth plasticity index (PI) across differing fertilizer concentrations (see methods for index calculation). Analysis was performed on trait means obtained from fixed-effect lsmean estimates from mixed models accounting for factors of non-interest (i.e., greenhouse block, cell culture plate). Mutualistic benefit was regressed against cell growth and the interaction between cell growth and fertilizer media treatments. Host genotype origin refers to the host genotype the isolate was isolated from during whole soil inoculations. Host genotype was included as genotype was identified as potentially important factor based on results of other components of this study. Non-significant interactive effects with Host genotype were excluded from the final model. [file peerj-03-1291-s004.docx]

Table S4: Analysis of genetic correlations between host partner quality (estimated from total fruit and flower production) and in vitro cell growth responses using mean rhizobia isolate values. Rhizobia growth responses were measured using optical density readings (OD_600_) within each fertilizer concentration or a growth plasticity index (PI) across differing fertilizer concentrations (see methods for index calculation). Analysis was performed on trait means obtained from fixed-effect lsmean estimates from mixed models accounting for factors of non-interest (i.e. greenhouse block, cell culture plate). Mutualistic benefit was regressed against cell growth and the interaction between cell growth and fertilizer media treatments. Host genotype origin refers to the host genotype the isolate was isolated from during whole soil inoculations. Host genotype was included as genotype was identified as potentially important factor based on results of other components of this study. Non-significant interactive effects with Host genotype were excluded from the final model.

| Fixed Effects | F (NumDF, DenDF) | P |
| --- | --- | --- |
|  |  |  |
| OD_600_ in no fertilizer |  |  |
| Field Fertilization (FF) | 2.21 (1, 42) | 0.1531 |
| Cell Density (OD_600_) | 0.09 (1,42) | 0.7638 |
| FF*Cell Density | 2.01 (1,42) | 0.1639 |
| Genotype | 0.90 (2,42) | 0.4149 |
|  |  |  |
| OD_600_ in low fertilizer |  |  |
| Field Fertilization (FF) | 2.10 (1, 43) | 0.1549 |
| Cell Density (OD_600_) | <0.00 (1,43) | 0.9918 |
| FF*Cell Density | 2.47 (1,43) | 0.1235 |
| Genotype | 0.70 (2,43) | 0.504 |
|  |  |  |
| OD_600_ in high fertilizer |  |  |
| Field Fertilization (FF) | 0.02 (1, 43) | 0.8905 |
| Cell Density (OD_600_) | 0.29 (1,43) | 0.5931 |
| FF*Cell Density | 0.01 (1,43) | 0.9348 |
| Genotype | 0.66 (2,43) | 0.5211 |
|  |  |  |
| Plasticity Index, low fertilizer |  |  |
| Field Fertilization (FF) | 4.50 (1, 43) | 0.0397 |
| Plasticity Index (PI) | 0.01 (1,43) | 0.9205 |
| FF*PI | 6.25 (1,43) | 0.0163 |
| Genotype | 1.31 (2,43) | 0.2815 |
|  |  |  |
| Plasticity Index, high fertilizer |  |  |
| Field Fertilization (FF) | 1.27 (1, 43) | 0.2653 |
| Plasticity Index (PI) | 0.01 (1,43) | 0.9221 |
| FF*PI | 1.75 (1,43) | 0.1927 |
| Genotype | 0.93 (2,43) | 0.4032 |
